# Dual-Task Gait and Balance Training Integrated With Sensory-Motor Interventions for Children With Autism Spectrum Disorder: A Comprehensive Narrative Review

**DOI:** 10.7759/cureus.93268

**Published:** 2025-09-26

**Authors:** Maheshkumar Baladaniya, Shraddha Baldania, Anamitra Hait, Arbind Kumar Choudhary

**Affiliations:** 1 Physical Therapy and Rehabilitation, Neighborhood Physical Therapy PC, New York, USA; 2 Department of Physical Therapy, Enjoy Rehab PT PC, New York, USA; 3 Medicine, KG Hospital, Chittaranjan, IND; 4 Pharmacology, Government Erode Medical College and Hospital, Erode, IND

**Keywords:** autism spectrum disorder, balance, dual-task training, gait, neuroplasticity, pediatric rehabilitation, sensory-motor integration, virtual reality

## Abstract

Motor impairments, common in children with Autism Spectrum Disorder (ASD), significantly affect their developmental trajectories, impacting balance, coordination, and gait. Traditional interventions often fail to address the interconnected motor, cognitive, and sensory challenges in ASD. This narrative review explores the efficacy of integrated dual-task gait and balance training combined with sensory-motor interventions, emphasizing their potential to enhance functional outcomes in children with ASD. Following the Scale for the Assessment of Narrative Review Articles (SANRA) and Preferred Reporting Items for Systematic Reviews and Meta-analyses (PRISMA) frameworks, a comprehensive literature search was conducted on PubMed, ScienceDirect, and Google Scholar from 2010 to June 2025. The keywords with Boolean operators consisted of “autism spectrum disorder”, AND “dual-task training”, AND “sensory motor integration”, AND “gait training”, OR “balance training”, OR “motor coordination”. The studies reported that integrated dual-task and sensory-motor interventions significantly improve motor proficiency, postural control, and dynamic balance, with measurable gains in standardized assessments. Technology-enhanced modalities, including virtual reality and wearable sensors, enhance engagement and enable personalized progression, fostering neuroplasticity and functional transfer to daily activities. Benefits extend to attention regulation and executive function, supported by neuroimaging evidence of enhanced prefrontal cortical activation. However, challenges include therapist training, resource limitations, and insurance barriers, necessitating innovative solutions like scalable technology and telehealth. Future research should prioritize large-scale trials, standardized measures, and long-term follow-up to optimize protocols and support widespread clinical adoption.

## Introduction and background

Motor difficulties are a pervasive yet under-recognized domain of Autism Spectrum Disorder (ASD), affecting up to 90% of children and profoundly shaping their developmental trajectories [1]. Traditional approaches that isolate motor skills or sensory processes fail to address the complex and interwoven nature of motor, cognitive, and sensory challenges in ASD [2]. Children with ASD typically exhibit gait deviations, such as reduced stride length and increased base of support, which can be addressed through targeted intervention. Understanding these gait patterns is essential for developing effective treatment plans that incorporate sensory-motor strategies [3]. To harness neuroplasticity and provide context-rich, functionally relevant therapy, this review lays the groundwork for a paradigm shift that combines dual-task gait and balance training with sensory-motor interventions.

Motor coordination impairments appear in 50-95% of children with ASD and frequently co-occur with developmental coordination disorder, despite the core diagnostic criteria emphasizing social communication and restricted behaviors [4]. These issues emerge in early childhood and persist into adolescence, impairing autonomy and quality of life. They encompass fine motor delays, atypical gait, and poor balance [4,5]. Such motor deficits cascade across multiple domains, undermining academic performance through challenges like legible handwriting and participation in physical education, hindering social integration in activities such as playground games and team sports, compromising daily living skills including self-care and household chores, exacerbating mental health issues like elevated anxiety and reduced self-esteem, and straining family dynamics by increasing caregiver stress and promoting social withdrawal [4].

Emerging neuroimaging and neurophysiological studies have illuminated the underlying mechanisms of motor dysfunction in ASD [6]. These include cerebellar-cortical connectivity deficits that impair timing and motor learning, basal ganglia dysfunction that hampers movement initiation and sequencing, mirror neuron system anomalies that disrupt imitation and social motor learning, and executive network underconnectivity that limits planning and attentional control [6]. Additionally, temporal processing deficiencies and impaired sensory gating contribute to multisensory processing breakdowns, where vestibular, proprioceptive, and visual signals fail to integrate coherently, undermining anticipatory control and adaptive motor responses [7]. These neurobiological deficits underpin the motor and sensory challenges observed in ASD, necessitating interventions that address both motor coordination and sensory integration.

Integrating dual-task training with sensory-motor strategies draws on several foundational principles to address these challenges effectively [8]. These encompass neuroplasticity principles that emphasize activity-dependent synaptic remodeling, motor learning theory that advocates for variable practice and contextual interference, attention theory that leverages dual-task paradigms to foster automaticity, and dynamic systems theory that views motor behavior as emergent from interactions among the individual, task, and environment [8]. Naturalistic activities inherently demand concurrent cognitive and sensory-motor engagement in the brain. By coupling sensory inputs with motor-cognitive challenges, this approach mirrors developmental processes, promotes transfer to everyday functioning, and benefits from emerging technologies such as virtual/augmented reality, biofeedback, and wearable sensors for personalized, engaging interventions [8]. Hence, the objective of this narrative review was to evaluate the efficacy of integrated dual-task gait and balance training combined with sensory-motor interventions in improving motor proficiency, postural control, and dynamic balance in children with ASD, with secondary consideration of technology-enhanced approaches, clinical implications, and implementation challenges.

## Review

**Figure 1:**
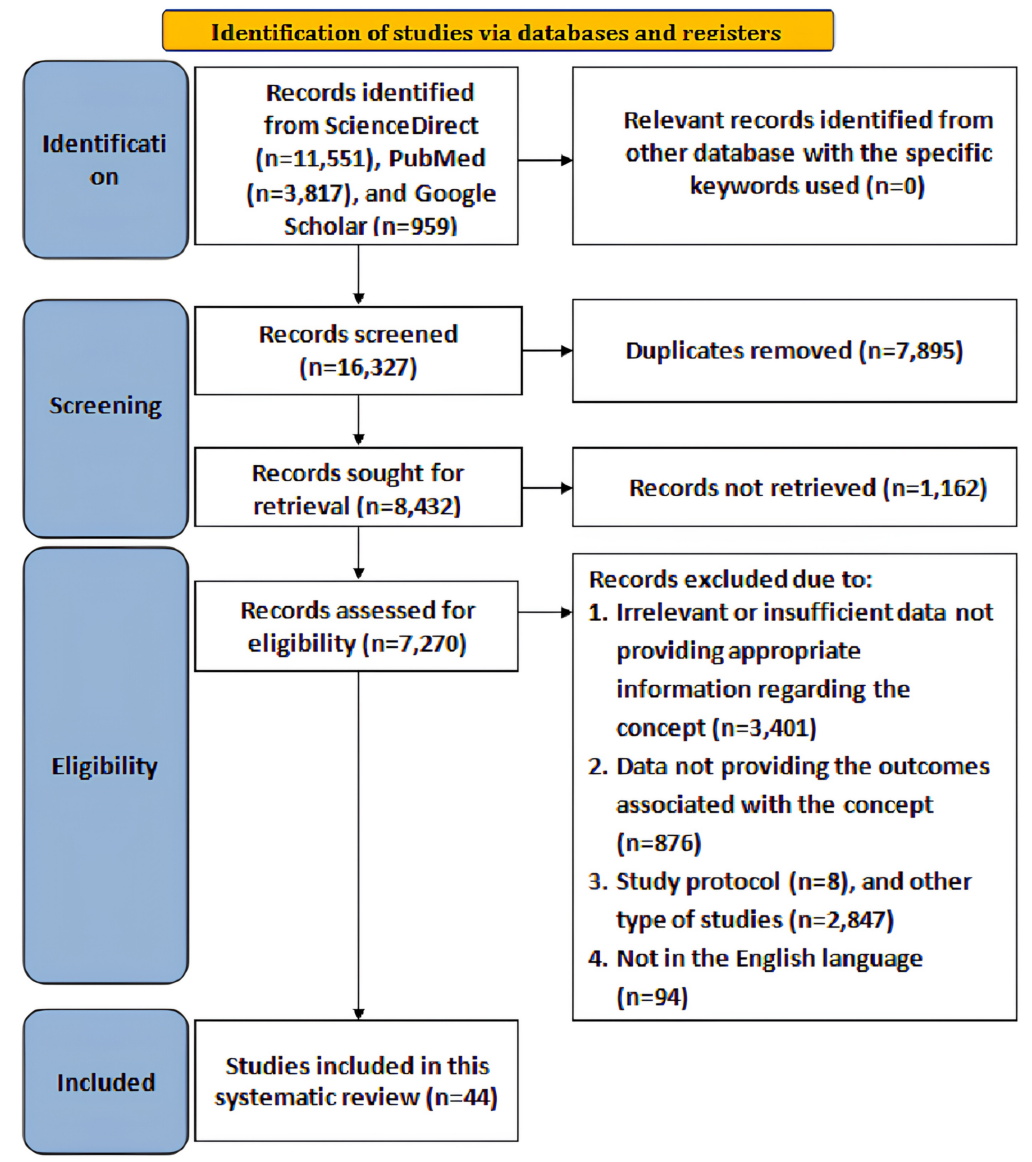
Search strategy

The Scale for the Assessment of Narrative Review Articles (SANRA) guided article selection, inclusion/exclusion criteria, critical appraisal, and evidence presentation [9] along with the Preferred Reporting Items for Systematic Reviews and Meta-analyses (PRISMA) framework [10], as illustrated in Figure 1.

Data sources and search strategy

A thorough literature search was conducted on PubMed, ScienceDirect, and Google Scholar databases from 2010 to June 2025, incorporating keywords with Boolean operators that consisted of “autism spectrum disorder”, AND “dual-task training”, AND “sensory motor integration”, AND “gait training”, OR “balance training”, OR “motor coordination”. The framed research question for the review was, "What is the efficacy of dual-task gait and balance training integrated with sensory-motor interventions in improving motor proficiency, postural control, dynamic balance, and functional outcomes in children with autism spectrum disorder (ASD)?"

Study screening and selection

The inclusion and exclusion criteria for the selection of studies are encompassed in Table 1.

**Table 1:** Inclusion and exclusion criteria for the selection of studies ASD = Autism Spectrum Disorder

Criteria Type	Criteria
Inclusion Criteria	1. Studies involving children aged 4–18 years with a confirmed ASD diagnosis.
	2. Interventions focusing on dual-task paradigms (motor-cognitive combinations), sensory-motor integration (vestibular, proprioceptive, tactile), or technology-enhanced approaches (virtual reality, biofeedback, wearables).
	3. Outcomes related to motor function, balance, attention, executive function, or functional independence.
	4. Study designs including randomized controlled trials, quasi-experimental studies, case series, systematic reviews, and meta-analyses.
Exclusion Criteria	1. Studies on adults or non-ASD populations.
	2. Interventions lacking an integrated dual-task or sensory-motor component.
	3. Articles with other study designs consisting of book chapters, opinion articles, and letters to the editor.
	4. Duplicates or inaccessible full texts.
	5. Studies lacking an available English translation or not published in English, with only a title and no abstract, those without open access, and those lacking sufficient knowledge with respect to the context.

Four reviewers independently assessed article eligibility for inclusion. To remove duplicates, titles and abstracts were screened initially, followed by a second review to exclude ineligible studies and a full-text assessment to confirm inclusion. Reviewer disagreements were resolved through discussion and consensus. To synthesize the findings, a critical narrative approach was employed [9]. The studies employed diverse methodologies and outcome measures, resulting in substantial heterogeneity.

Data extraction and synthesis

The data was extracted based on information that concentrated on assessing the impact of dual-task gait and balance training integrated with sensory-motor interventions, technology-enhanced intervention, clinical outcomes, functional impact, implementation considerations, clinical practice and implications, challenges and solutions, and future directions for children with ASD. The critical narrative technique was utilized to incorporate figures and text to summarize and validate evidence.

Dual-task training approaches: evidence and applications

The current literature reveals a growing interest in dual-task methodologies that combine motor activities (such as walking and balance tasks) with cognitive challenges (working memory, verbal fluency, and attention tasks). Clinical investigations have shown that children with ASD can successfully engage in dual-task paradigms, although initial performance may be reduced due to cognitive load competition during early learning phases. For instance, dual-task treadmill training has been shown to improve axial and proximal motor skills in children with ASD in a quantifiable manner [11,12]. However, adaptation phases vary across studies due to differences in intervention duration and methodology, and systematic comparisons of these phases are limited, highlighting the need for future research to standardize and explore adaptation timelines. Moreover, because of the brief intervention period (four weeks) or the intrinsic complexity of motor learning in ASD populations, changes in particular gait parameters might not achieve statistical significance over brief intervention periods. According to these results, children with ASD require longer intervention times for significant functional improvements because their motor adaptation occurs on a different schedule than that of neurotypical development [11]. 

A comparative study examining single-task versus dual-task balance training has revealed interesting patterns in functional outcomes. Yaghoubi et al. found that both single- and dual-task balance exercises improved functional balance, although dual-task approaches showed advantages in specific measures, such as the Pediatric Berg Balance Scale [12]. The modest effect sizes observed may reflect the early learning phase, where cognitive load temporarily reduces motor performance, suggesting that longer intervention periods may be necessary to achieve automatization and larger effect sizes [12]. Although the intricacy of these interventions necessitates careful individualization based on cognitive capacity and motor skill level, it was observed that dual-task training improves neuroplasticity by utilizing shared neural resources for attention and motor control [11,12].

Sensory-motor integration: addressing core deficits

The literature emphasizes that difficulties in sensory processing are fundamental to motor challenges in ASD, requiring targeted interventions that simultaneously address vestibular, proprioceptive, and tactile integration [13]. Research has consistently demonstrated the effectiveness of sensory integration approaches in improving both motor function and broader developmental outcomes [13,14]. According to clinical studies, children with ASD benefit greatly from sensory integration therapy (SIT), which incorporates vestibular and proprioceptive stimulation, in terms of postural control and balance. After organized multi-month interventions, studies show significant decreases in postural sway measures (up to 25% improvement) [2,15]. Functional near-infrared spectroscopy (fNIRS) neurophysiological evaluations show that SIT improves executive function and motor performance, with dorsolateral prefrontal cortex areas linked to cognitive control showing greater activation [2,13].

Balance training programs incorporating sensory integration techniques have demonstrated improved postural control and confidence in children with ASD, as evidenced by significant reductions in postural sway measures following multi-month interventions [15]. A systematic review of postural balance control interventions indicates that sensory integration training significantly enhances balance, particularly under challenging visual conditions [16]. Children with ASD can benefit greatly from balance interventions that address sensory integration issues [2]. Studies consistently reported significant improvements in balance skills after the intervention, and a meta-analysis of balance-focused interventions found large positive effects [2,17]. These results underline how important sensory-motor integration is in addressing the complex motor difficulties that are a feature of ASD.

Research has explored diverse approaches to balance training within sensory-motor integration frameworks for children with ASD, emphasizing activities that combine motor challenges with sensory processing to enhance functional outcomes. For example, martial arts interventions, such as Taekwondo training, have demonstrated significant improvements in balance, as evidenced by enhanced performance on balance-specific measures in children with ASD [18]. Similarly, structured Motor Sensory Room training, which provides specialized environments to stimulate vestibular, proprioceptive, and tactile systems, has shown significant improvements in sensory integration profiles and motor skills, as measured by the Movement Assessment Battery for Children (M-ABC-2) balance scores [19,20]. These approaches, by integrating sensory-rich environments with motor tasks, align with sensory-motor integration principles and demonstrate the potential of varied, context-rich interventions to enhance balance and coordination, supporting broader developmental gains in children with ASD. These improvements in motor coordination and relational behaviors seem to hold up over follow-up times, indicating that intensive sensory-motor integration techniques could result in long-lasting functional gains. The literature reveals that various physical therapy techniques, including gross motor development strategies and sensory-motor integration, effectively enhance motor planning and coordination in children with ASD [21]. Physiotherapy techniques such as rhythmic activities and multimodal approaches have been effective in improving coordination and balance in children with ASD, demonstrating positive effects on motor proficiency and social interaction [22].

Technology-enhanced interventions: innovation in practice

The literature increasingly highlights virtual reality (VR) as a transformative tool for pediatric ASD interventions. Research demonstrates that VR-based training can effectively improve postural balance in children with autism spectrum disorder through randomized controlled trials [23,24]. Immersion environments that boost motivation and enable precise task difficulty modulation are offered by virtual reality and treadmill-based dual-task scenarios [25]. Non-wearable VR platforms provide immersive environments where children can practice dual-task activities while receiving real-time feedback and adaptive challenge progression [26,27]. VR-based behavioral training supports improvements in cognitive abilities, motor skills, sensory processing, and response inhibition, with sustained gains over several months post-intervention, according to clinical protocols aimed at preschoolers with high-functioning ASD [27,28]. Research indicates that VR systems effectively address the sensory overload and anxiety often experienced by children with ASD by providing controlled, customizable virtual environments [29,30]. Hence, the integration of virtual reality and artificial intelligence in autism therapy represents a promising frontier for personalized intervention delivery [31].

Clinical investigations demonstrate that wearable devices can effectively assess gait and physical activity in various conditions, enabling precise monitoring of motor performance during training sessions [32-34]. These technologies facilitate individualized progression through real-time performance feedback and objective outcome measurement, supporting evidence-based treatment modification. Wearable sensors and biofeedback systems enable real-time monitoring and personalized adjustment of training programs, facilitating progression into automated performance modes [32]. Wearables equipped with accelerometers and motion-capture capabilities enable continuous monitoring of gait, balance, and physical activity during training and daily life [32]. Hence, healthcare monitoring systems using Internet of Things (IoT) platforms provide comprehensive tracking capabilities for intervention outcomes [34]. Research suggests that technology-enhanced interventions can support neuroplastic changes in brain networks governing attention and motor control, as neurofeedback training can normalize behavioral and electrophysiological measures in high-functioning autism [35]. The integration of sophisticated monitoring and feedback systems into dual-task and sensory-motor training programs is supported by these findings.

Clinical outcomes and functional impact

Literature synthesis reveals consistent patterns of improvement in motor proficiency, postural control, and dynamic balance following integrated dual-task and sensory-motor interventions, despite heterogeneity in study designs, including randomized controlled trials (RCTs), quasi-experimental studies, case series, and systematic reviews [36]. While these diverse designs contribute to a robust overall picture, they vary in evidence level, and this review does not stratify findings by study design rigor due to its narrative approach, which prioritizes thematic synthesis over hierarchical evidence ranking. Clinical studies consistently report enhanced performance on standardized assessments, such as the Movement Assessment Battery for Children (M-ABC-2) and pediatric balance scales [15,20]. Moreover, studies demonstrated notable gains in both static and dynamic balance skills, as well as decreased postural sway and increased self-assurance when performing difficult balancing tasks [15,37,38]. Measurable gains in motor coordination and postural control have been demonstrated by physical activity designs that specifically target balance rehabilitation in children with ASD [15].

Research indicates that integrated motor-cognitive training produces benefits extending beyond motor skills, with improvements observed in attention regulation, executive function, and behavioral organization. Neuroimaging studies using fNIRS technology suggest enhanced activation in prefrontal cortical regions associated with cognitive control and executive function [2]. These findings support the interconnected nature of motor and cognitive development, highlighting the advantages of integrated intervention approaches.

When compared to isolated interventions, integrated training approaches may result in better transfer to everyday activities, according to parent reports and clinical observations [39]. Improvements in handwriting skills, playground participation, and community navigation have been reported by parents and clinicians, though primarily based on observational reports rather than validated assessment tools [40,41]. The lack of systematic documentation using standardized measures for these functional outcomes highlights a critical area for future research to strengthen the evidence base. Hence, context-rich interventions that replicate real-world problems exhibit superior generalization compared to isolated skill training conducted in a traditional clinic.

Implementation considerations and clinical practice

The literature consistently emphasizes the heterogeneity of ASD populations and the critical need for personalized intervention planning. Research suggests that intervention effectiveness depends on the appropriate matching of dual-task complexity, sensory inputs, and motor challenges to individual cognitive and motor capabilities [12]. Clinical studies indicate that children across different ASD severity levels can benefit from integrated approaches, though intervention parameters require careful adaptation. Current evidence suggests that longer intervention periods (≥12 weeks) and higher training frequencies (≥3 sessions per week) may be necessary to achieve meaningful functional improvements in children with ASD [42,43]. However, optimal dosing parameters, including session duration, intervention intensity, and progression strategies, remain areas requiring systematic investigation [42,43]. The literature indicates that motor learning in ASD populations may require extended practice periods to achieve automatization and functional transfer [44].

Value-based pricing frameworks that prioritize long-term cost savings and functional outcomes, as well as digitally enabled care models, must be taken into account when implementing innovative interventions in the healthcare system [37,45]. For sophisticated dual-task and sensory-motor interventions to be successfully incorporated into standard clinical practice, these factors are crucial. Research emphasizes the importance of coordinated care involving physical therapists, occupational therapists, speech-language pathologists, and other specialists to address the multifaceted nature of ASD-related challenges [38]. Hence, the literature supports community-based integration models that incorporate dual-task training in school physical education programs and community centers, fostering naturalistic practice and peer interactions.

Telehealth platforms and remote delivery models show promise for overcoming geographical barriers and expanding access to specialized interventions [46]. Parent-mediated coaching and remote monitoring systems facilitate sustained engagement and continuity of care across settings. Current advancements in autism therapy emphasize the integration of artificial intelligence, virtual reality technologies, wearable devices, precision medicine approaches, and telehealth delivery models [47]. These innovations represent the cutting edge of intervention development and hold promise for transforming clinical practice in pediatric ASD rehabilitation.

Clinical implications

Recent advances in integrated dual-task gait and balance training combined with sensory-motor interventions for children with ASD emphasize the transformative potential of emerging technologies and novel program models. Physiotherapy techniques, such as rhythmic activities and multimodal approaches, have been effective in improving coordination and balance in children with ASD [22]. These interventions have demonstrated positive effects on motor proficiency and social interaction, which are crucial for daily functioning [22].

Virtual reality (VR) and augmented reality (AR) applications have rapidly evolved into promising tools for ASD interventions, particularly in preschool-and school-aged children. Non-wearable VR platforms with immersive and interactive environments enable children to engage in complex dual-task gait and balance training with simultaneous cognitive challenges that mimic real-world, multisensory scenarios. These VR systems adapt difficulty in real-time based on performance, sustain motivation, and optimize challenge levels [23,26]. For instance, behavioral training using non-wearable VR technology, including interactive floor projections, real-time monitoring, and cloud-data management, was integrated into a large randomized controlled trial protocol for preschoolers with high-functioning ASD (ages 4-7). This intervention reduces sensory overload and anxiety by allowing children to complete simultaneous motor and cognitive tasks in visually rich scenarios customized to their sensory and cognitive profiles. Preliminary data indicate that VR behavioral training promotes gains in motor skills, response inhibition, cognitive abilities, and sensory processing that persist for several months post-intervention [27].

Moreover, VR facilitates social interaction through multiplayer modes and teletherapy capabilities, expanding access and reducing therapy costs. Controlled, customizable virtual environments provide safe spaces for practicing life skills and social communication while minimizing real-world social anxiety in children with ASD [27,29,30]. Artificial intelligence integration further enhances these systems, with AI-powered adaptive training automatically adjusting task difficulty based on continuous performance feedback to optimize individual challenges and progression. Predictive analytics and pattern recognition support personalized program design and real-time feedback delivery, fostering neuroplastic changes in brain networks governing attention and motor control [35]. Wearable technology and the Internet of Things (IoT) complement these advancements, as wearables with accelerometers and motion-capture capabilities enable continuous monitoring of gait, balance, and physical activity during training and daily life. These data streams support home-based programs with remote supervision, bidirectional feedback, and family connectivity through apps, promoting sustained engagement and objective outcome tracking [33].

Challenges and solutions

Integrated dual-task gait and balance training combined with sensory-motor interventions presents several challenges in clinical practice; however, targeted solutions and innovations can address them effectively [2]. One key challenge is therapist training and competency, as these approaches require advanced skills in motor function, sensory integration, cognitive dual-tasking, and technology use, areas where many physical therapists and clinicians lack specialized training. To overcome this, the development of specialized certifications and continuing education programs focused on integrated motor-sensory-cognitive therapies for ASD is essential. Innovative training modalities, such as virtual reality-based therapist simulations and mentoring systems, can accelerate skill acquisition and improve competence. For example, VR training programs allow therapists to practice intervention delivery in immersive, risk-free environments, enhancing learning efficiency [31].

Resource and equipment needs pose another significant barrier, as advanced interventions often rely on technological equipment such as VR systems, wearable sensors, and biofeedback devices, along with appropriate space, which may be cost-prohibitive or inaccessible in some settings [31,34]. Scalable intervention models that utilize shared or affordable equipment, such as low-cost wearable sensors or community-shared VR systems, can expand access [34,45]. Additionally, mobile intervention units and pop-up therapy programs, inspired by successful mobile health and tele-rehabilitation models for underserved populations, can reduce geographical barriers by delivering specialized interventions to remote or resource-limited areas [46,48]. These approaches leverage existing telehealth frameworks and portable technology to enhance accessibility, as demonstrated in telerehabilitation programs for ASD that improve access to care [46]. Innovations such as smartphone applications linked with wearable sensors enable remote monitoring and home-based training, reducing dependency on clinic-based resources while maintaining quality [35,46].

Insurance and funding barriers further complicate adoption, as many innovative interventions lack formal recognition or reimbursement codes in health insurance systems. Systematic documentation of clinical outcomes, cost-effectiveness data, and patient-reported benefits will support advocacy for coverage, with collaboration among researchers, advocacy groups, and payers driving policy changes. Value-based care models emphasizing functional improvements and long-term cost savings can integrate these approaches into mainstream reimbursement frameworks [37].

Future directions

**Figure 2:**
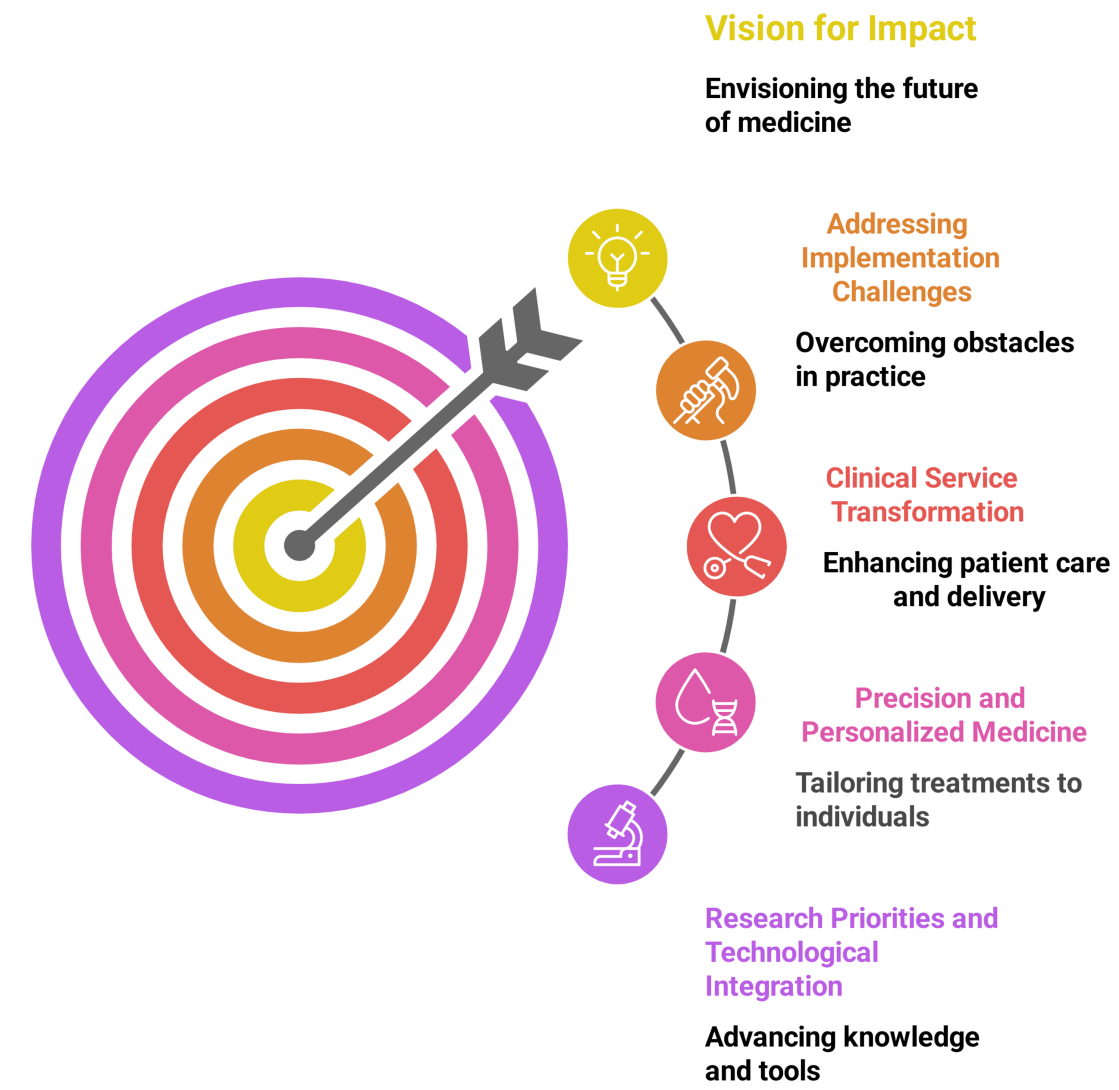
Future directions for dual-task gait and balance training integrated with sensory-motor interventions The figure is created by the authors illustrating future directions for dual-task gait and balance training integrated with sensory-motor interventions.

Building on the comprehensive understanding of dual-task gait and balance training integrated with sensory-motor interventions for children with ASD, the future trajectory in this field is poised to leverage technological innovation, precision medicine, and expanded service delivery models to enhance outcomes, accessibility, and personalization, as illustrated in Figure 2.

Research priorities and technological integration

Artificial intelligence and machine learning will play a pivotal role, with AI-driven adaptive training systems and algorithms optimizing individualized intervention doses, continuously tracking progress, and predicting treatment responsiveness based on neurophysiological and behavioral data [49,50]. This will enable precision-tailored programs that evolve dynamically with a child's capabilities and needs. Virtual and augmented reality platforms will continue to expand, becoming more immersive, interactive, and socially integrative, providing realistic dual-task environments that safely simulate complex real-world scenarios. These tools will support cognitive-motor integration, social communication, and sensory regulation in engaging, customizable contexts [51]. Advances in wearable sensors and Internet of Things (IoT) connectivity will facilitate round-the-clock monitoring of motor behavior, balance, and physiological states, enabling real-time feedback and remote interventions. Family and clinician dashboards will enhance collaborative care and treatment adherence [52].

Precision and personalized medicine

Future research should focus on identifying neurobiological and genetic biomarkers that predict a response to integrated motor-cognitive-sensory interventions, enabling personalized therapy matching and offering mechanistic insights into neuroplastic changes. Large datasets should be used to develop predictive algorithms for outcomes based on age, ASD severity, cognitive profile, and sensory integration status, improving intervention selection and timing. Adaptive protocols should increasingly use real-time data to modify complexity, sensory inputs, and dual-task demands, optimizing neuroplasticity and functional gains while minimizing cognitive overload. Continued longitudinal research is necessary to assess the long-term benefits of sensory-motor interventions, particularly across diverse populations and varying ASD severity levels [20]. Additionally, research should prioritize equity in access to interventions, ensuring that diverse populations, including those from underserved communities, benefit from tailored programs. Cultural and contextual adaptations of interventions, such as incorporating culturally relevant activities or addressing language and socioeconomic barriers, are critical to ensure inclusivity and effectiveness across varied demographic groups [53]. Interdisciplinary approaches integrating physical therapy with other disciplines promise to address the multifaceted needs of children with ASD more effectively [38]. Overcoming variability in therapeutic outcomes and expanding access to specialized services will be the next critical steps.

Clinical service transformation

Integrated multidisciplinary care models will foster greater collaboration among physical therapists, occupational therapists, speech-language pathologists, psychologists, educators, and families, forming comprehensive teams focused on holistic child development. Community and school-based programs will integrate dual-task sensory-motor training into curricula and recreation activities, enhancing naturalistic practice, peer engagement, and skill generalization. To promote equity, these programs should be designed to reach underserved populations through culturally sensitive adaptations, such as integrating community-specific recreational activities or providing multilingual resources to address diverse needs [53]. Telehealth and remote delivery platforms will expand access, especially for remote or underserved areas, by combining VR/AR interventions with caregiver coaching and digital monitoring. Recent systematic reviews demonstrate that telerehabilitation significantly improves communication skills and social functioning in children with ASD, with parent-mediated programs showing high effectiveness and satisfaction rates [54].

Addressing implementation challenges

To ensure the successful integration of dual-task gait and balance training combined with sensory-motor interventions into clinical practice, targeted strategies must address key barriers through both short-term actionable goals and long-term aspirational goals. Short-term actionable goals include extending intervention durations to achieve meaningful functional improvements (e.g., ≥12 weeks, ≥3 sessions per week), enhancing therapist training through specialized certifications and virtual reality-based simulations, and refining outcome measures by adopting standardized tools like the Movement Assessment Battery for Children (M-ABC-2) to improve consistency and comparability across studies [20,42,43]. These steps can be implemented promptly to enhance the efficacy of interventions and their clinical adoption. Long-term aspirational goals involve leveraging artificial intelligence (AI) and biomarker-driven personalization, such as developing AI-driven adaptive training systems and identifying neurobiological markers to optimize individualized intervention protocols [49,50]. These advancements require sustained research and technological development but promise transformative personalization. Additionally, equity-focused strategies, such as subsidized equipment programs or partnerships with community organizations, will be essential to reduce disparities in access to advanced interventions [48]. Robust clinical outcome data and health economics research will support insurance coverage and policy reforms for sustained advanced therapy [54,55].

Vision for impact

The next decade envisions a fundamental transformation in pediatric ASD motor rehabilitation through harnessing technological advancements to create engaging, precisely tailored, and effective interventions; embracing personalized approaches grounded in neurobiological understanding and adaptive delivery; and expanding accessibility and equity through remote care and community integration. This will ultimately improve meaningful functional independence, social participation, and quality of life for children with ASD worldwide. These future directions emphasize a comprehensive, data-informed continuum of care that integrates motor, sensory, and cognitive domains through state-of-the-art technology and interdisciplinary collaboration, aligning with 2025 trends in autism therapy innovation, including AI, VR/AR, wearables, precision medicine, and telehealth [47,56].

## Conclusions

This comprehensive review demonstrates that integrated dual-task gait and balance training combined with sensory-motor interventions represents a promising advancement in rehabilitation for children with ASD. The evidence synthesis indicates that multi-domain approaches targeting motor, cognitive, and sensory systems simultaneously yield superior outcomes compared to conventional single-domain therapies. Key findings include significant gains in dynamic balance, postural control, motor coordination, and attention regulation. Notably, these integrated approaches appear to better harness neuroplasticity mechanisms and exhibit improved transfer to real-world functional activities than isolated interventions. Technology-enhanced modalities, particularly virtual reality and biofeedback systems, show particular promise for enhancing engagement and facilitating personalized progression; however, more rigorous controlled trials are needed to refine optimal implementation protocols. Despite these encouraging findings, several important limitations must be acknowledged, including small sample sizes, short intervention durations, methodological heterogeneity, limited long-term follow-up data, heterogeneity of ASD severity, and lack of functional outcome standardization. Implementation barriers, including the need for specialized training, resource allocation, and insurance coverage restrictions, severely hamper widespread clinical adoption. Future research should prioritize large-scale randomized controlled trials (RCTs) with prolonged follow-up periods, standardized outcome measures, and systematic examination of dose-response relationships to strengthen the evidence base. Additionally, while innovations like AI, VR, precision medicine, and telehealth offer promising avenues for advancing intervention delivery, their speculative nature underscores the need for robust empirical validation to confirm their feasibility and effectiveness. Realizing the potential of this integrated paradigm to transform ASD motor rehabilitation will require sustained research funding, clinician education programs, and healthcare system reforms to support these multi-domain interventions in standard clinical practice.
